# Examining longitudinal associations between initial perceptions and experiences with electronic nicotine delivery system (ENDS) use and use patterns among adults who smoke and recently initiated ENDS

**DOI:** 10.18332/tid/193009

**Published:** 2024-09-27

**Authors:** Michelle Mavreles Ogrodnick, Nikita G. Kute, Vuong Van Do, Paige Wiley, Katherine Henderson, Claire A. Spears, Terry F. Pechacek, Scott R. Weaver

**Affiliations:** 1 Department of Population Health Sciences, School of Public Health, Georgia State University, Atlanta, United States; 2 Center for Tobacco Control Research and Education, University of California, San Francisco, United States; 3 Department of Health Policy and Behavioral Sciences, School of Public Health, Georgia State University, Atlanta, United States

**Keywords:** electronic nicotine delivery systems (ENDS), tobacco regulatory science, tobacco users, dual users

## Abstract

**INTRODUCTION:**

Limited data exist on factors associated with concurrent use patterns of electronic nicotine delivery systems (ENDS) and cigarettes. We examined longitudinally perceptions and experiences with ENDS in relationship to concurrent use patterns among established, recent smokers who recently initiated ENDS.

**METHODS:**

Participant recruitment took place using paid digital advertisements on social media. Between December 2020 and October 2021, 303 adults aged ≥21 years from across the US who currently or recently smoked and had initiated ENDS use within the past 30 days or reinitiated ENDS use after more than one year of non-use were surveyed. Multinomial logistic regressions were conducted to analyze association between the outcome of current use pattern at follow-up at 1 month [rejectors (discontinued ENDS, continued smoking), primary smokers (concurrent users, mostly smoke), dual user (similar smoking and ENDS use), primary vapers (concurrent users, mostly vape), and switchers (discontinued smoking, continued using ENDS) or quitters (discontinued both smoking and ENDS] and perceptions of and experiences with ENDS predictors at baseline.

**RESULTS:**

At follow-up at 1 to 2 months after initiating ENDS, 20% were rejectors, 31% were primary smokers, 13% were dual users, 19% were primary vapers, and 17% were switchers/quitters. Perceiving ENDS as less harmful than smoking or being uncertain and as equally or more enjoyable smoking, experiencing a lot or complete reduction in cravings to smoking and in irritability with ENDS use, liking the taste of ENDS, and being satisfied with vaping were associated with higher odds of quitting smoking compared to rejecting ENDS or mostly smoking at follow-up at 1 month.

**CONCLUSIONS:**

Findings highlight the importance of initial ENDS perceptions and experiences when examining tobacco outcomes and potentially for developing policies and interventions targeting smoking cessation. ENDS initiators are differentiating into distinct use patterns based on these factors within a short period of time.

## INTRODUCTION

Recent data indicate concurrent use of both cigarettes and ENDS remains relatively common^1,2^. Approximately 31.4% of people who are smoking reported using e-cigarettes in 2021^3^, and many smokers initiate ENDS use in the hope that these products will help them quit smoking cigarettes^4-6^. However, there is varied evidence on whether ENDS are effective as a smoking cessation tool under real-world conditions^1,7-10^. While exposure to carcinogenic toxicants is reduced when switching from exclusive cigarette use to exclusive ENDS use, evidence also shows that continued concurrent use of cigarettes and ENDS does not reduce the exposure significantly and may lead to adverse health outcomes^11,12^. According to the Centers for Disease Control and Prevention (CDC), ‘E-cigarettes may have the potential to benefit adult smokers who are not pregnant if used as a complete substitute for regular cigarettes and other smoked tobacco products’^13^. Research suggests that additional factors such as the enjoyment of ENDS flavors, social acceptability (e.g. being part of a peer group that uses ENDS and being able to vape indoors with peers), and the ability to manage negative perceptions like stigma and harm, may contribute to the concurrent use of ENDS and cigarettes^4,14^.

Among concurrent users, the most common patterns are to continue dual use or transition to exclusive cigarette smoking, while it is less common to transition to exclusive ENDS use^15,16^. Dual users often note that they did not completely switch to ENDS because ENDS did not meet their expectations^4,17^. For example, for some of them, ENDS did not provide the same flavors or the density of the smoke from ENDS was not similar to that from cigarettes, leading to a disappointing experience^4,18-20^. Other factors that may affect user expectations and deter them from smoking cessation are disliking the taste of ENDS products, finding ENDS unenjoyable, unsatisfying, and unable to manage cigarette cravings and irritability^21-23^.

A cigarette smoker’s initial experiences and perceptions with ENDS is likely to determine their continued use of ENDS, and whether ENDS substitute or complement their cigarette consumption^17^. However, limited research exists that examines the experiences or perceptions of smokers who recently initiated ENDS^1,17,24,25^. Understanding how initial ENDS use experiences such as taste, cigarette craving reduction, irritability reduction and initial perceptions of relative harm and enjoyability associate with use patterns could help identify pathways leading to sustained dual use or relapse to smoking, compared to completely switching to ENDS or quitting tobacco use which could have implications in policy, communications, and interventions^26^. In addition, understanding how people who smoke progress into different ENDS and cigarette use patterns with multiple ENDS products rapidly introduced in the market, can help determine if ENDS are really a disruptive technology that leads to a reduction in combustible tobacco use and ultimately reduces tobacco-related diseases and death^10,27^. Accordingly, this study aims to examine short-term cigarette and ENDS use outcomes among established cigarette smokers following ENDS initiation in relation to perceptions and experiences with ENDS. Whereas prior research has tended to classify concurrent users of cigarettes and ENDS monolithically, some recent research has further differentiated this group according to their relative frequency or amount of use, such as primary smokers, dual users, or primary vapers^6,16,25,28,29^. Our study will use these more informative categories of dual use to investigate the association of ENDS and cigarette use patterns with several relevant perceptions and experiences (i.e. perceived relative harm, enjoyment from ENDS, cigarette craving reduction from ENDS use, irritability reduction with ENDS use, ENDS satisfaction, and ENDS taste liking). We hypothesized that more positive initial experiences with ENDS (e.g. satisfaction, craving reduction) would be more common among switchers/quitters and primary vapers, compared to rejectors and primary smokers. By examining relevant perceptions and experiences with cigarettes and ENDS in the early period of initiating (or reinitiating) ENDS, we are better able to understand characteristics of the multiple different emerging use patterns.

## METHODS

### Sample and procedures

Participants were 303 US adults, aged ≥21 years who enrolled in the Adult Consumers of Tobacco Study (ACTS) following recruitment using paid digital advertisements on social/online media platforms (e.g. Facebook, Twitter, and Craigslist) during December 2020 to October 2021^30^. Eligible participants were adults who had an established, recent or current cigarette smoking history, defined as having smoked at least 100 cigarettes in one’s lifetime and either current smoking cigarettes on some days or every day or having smoked within the past 60 days, and recent initiation of ENDS, defined as having either tried an ENDS product for the first time within the past 30 days or reinitiated use of ENDS after a year or more break within 30 days of taking the eligibility survey. Individuals who clicked on the paid advertisement were directed to an online eligibility survey hosted on the Qualtrics platform. Those individuals whose responses indicated potential eligibility and passed multiple fraud detection and prevention checks, were prompted for their contact information. After passing subsequent additional fraud detection and prevention checks, they were invited by email and text message to take an initial baseline survey exactly seven days following completion of the eligibility survey. Individuals who completed the baseline survey were considered enrolled participants in the longitudinal study and automatically scheduled for subsequent weekly surveys, for which they had 48 hours following email/text invitation to begin. Data for this study come from the eligibility, baseline, and week 5 (follow-up at 1 month) surveys. Participants were compensated with a $20 e-gift card for completing the baseline survey and $10 for completing the week 5 survey. Informed consent for the eligibility survey and longitudinal study was collected, and the Georgia State University Institutional Review Board approved this study as exempt from ethical approval^30^.

### Measures

*Cigarettes and ENDS use status*

Our outcome variable was assessed at follow-up at 1 month using two questions measuring current cigarette use (‘Do you now smoke cigarettes every day, some days, or not at all?’ and ‘On how many of the past 7 days did you smoke cigarettes?’) and two questions measuring current ENDS use (‘Do you now use electronic nicotine products with nicotine every day, some days, or not at all?’ and ‘On how many of the past 7 days did you use an electronic nicotine product?’). Based on prior work, responses to these four questions were used to compute the outcome variable classifying participants into one of five use patterns: 1) rejectors, 2) primary smokers, 3) dual users, 4) primary vapers, and 5) switchers/quitters^27,28^. Rejectors reported currently smoking cigarettes but not ENDS. Primary smokers reported either smoking cigarettes every day and using ENDS on some days or smoking both cigarettes and using ENDS some days but with greater number of cigarette smoking days than ENDS use days in the past week. Dual users reported either both smoking cigarettes and using ENDS every day or both smoking cigarettes and using ENDS on some days with an equal number of smoking and ENDS use days in the past week. Primary vapers reported either using ENDS every day and smoking some days or using ENDS and smoking on some days with a greater number of ENDS use days than smoking days in the past week. Switchers were participants who reported using ENDS but not cigarettes every day or some days, whereas quitters reported they were not currently smoking or using ENDS. Due to the limited number of quitters, they were combined into one group with the switchers for the analyses.

*Predictor variables*

Predictor variables consisted of several ENDS perception and experience variables. Perceived relative risk of ENDS was assessed by: ‘Is using electronic nicotine products less harmful, about the same, or more harmful than smoking regular cigarettes?’ with five response options ranging from ‘much less harmful’ to ‘much more harmful’, as well as ‘don't know’, collapsed to ‘less harmful’ vs ‘equally or more harmful’ vs ‘don't know’. Relative enjoyability of ENDS compared to cigarettes was measured by: ‘How would you compare the experience of using electronic nicotine products to smoking regular cigarettes?’ with response options ‘electronic nicotine products are more enjoyable’, ‘equally enjoyable’, or ‘electronic nicotine products are less enjoyable’. For the analyses, this variable was dichotomized as ‘less enjoyable’ vs ‘equally or more enjoyable’. Cigarette craving reduction was assessed with: ‘When I use electronic nicotine products, my cravings to smoke a cigarette are reduced…’, with response options ‘not applicable’, ‘I do not have cravings to smoke a cigarette’ (coded as missing data), ‘not all’, ‘a little’, or ‘completely’. For the analyses, this variable was dichotomized as ‘not at all or a little’ vs ‘completely’. Irritability reduction when using ENDS (‘Did vaping make you feel less irritable?’), liking ENDS taste (‘Did your electronic nicotine product taste good?’), and satisfaction with using ENDS (‘Was vaping satisfying?’) were assessed on a seven-point scale ranging from 1= ‘not at all’ to 7 = ‘extremely’. When answering these questions, participants were asked to think about their typical experience when vaping.

*Covariates*

Assessed sociodemographic variables included age, gender identification (male/female), race/ethnicity (dichotomized as racial/ethnic minoritized group or non-Hispanic White), and education level (at least some post-secondary education vs secondary education or lower). Irritability reduction when smoking, liking the taste of cigarettes, and satisfaction with cigarettes were assessed with: ‘Did smoking make you feel less irritable?’, ‘Did the cigarette taste good?’ and ‘Was smoking satisfying?’ with the prompt to think about their typical experiences when smoking cigarettes. Cigarette withdrawal symptoms were assessed with four items from the Wisconsin Withdrawal Scale’s Urge subscale based on the item: Over the last 24 hours, I have: 1) had frequent urges to smoke, 2) been bothered by the desire to smoke a cigarette, 3) thought about smoking a lot, and 4) trouble getting cigarettes off my mind^31^. Response options were on a five-point Likert agreement scale. Item scores were averaged with higher scores denoting greater withdrawal. Psychological distress was assessed with the Kessler-6 scale with higher scores denoting greater psychological distress^32^.

### Statistical analysis

Descriptive statistics (proportions, means, standard deviations) were obtained for all variables. Multinomial logistic regression of cigarette and ENDS use status regressed on each ENDS perception and experience predictor and covariates was conducted. Due to collinearity among the predictors, separate regression models were conducted for each predictor. Analyses were conducted with Mplus software (v.8.4) using robust full-information maximum likelihood with Monte Carlo numerical integration. This approach allows for inclusion of all cases, including cases with some missing data, under the missingness at random (MAR) assumption, which is less stringent than the missing completely at random assumption of complete case analysis. Nine participants were excluded from the analyses due to missing data on one or more covariates, and for some analyses, one additional participant was excluded due to missing data on the primary predictor and outcome variables for an analytic sample size of 293 to 294. Covariates were considered exogenous in the models, while predictor variables of interest and the outcome variable were considered endogenous. A p<0.05 was considered statistically significant.

## RESULTS

Participant characteristics and descriptive statistics for study variables are reported in Table 1 and in Supplementary file Table S1. Approximately 66% of participants were female, about 63% had more than a high school degree, and about 73% were non-Hispanic White. At baseline, approximately 11% of participants were switchers/quitters, 19% primary vapers, 19% dual users, 45% primary smokers, and 6% rejectors. At follow-up at 1 month, 17% were switchers/quitters, 19% primary vapers, 13% dual users, 31% primary smokers, and 20% rejectors.

**Table 1:** Descriptive statistics for study variables among recent initiators of ENDS, 2020–2021 (N=303)

*Variables*	*n*	*Proportion*
**Tobacco user group** (at follow-up at 1 month)		
Exclusive smokers (rejectors)	54	0.20
Primary smokers	83	0.31
Dual users	35	0.13
Primary vapers	51	0.19
Switchers/quitters	45	0.17
**Perceived relative harm of ENDS vs cigarettes**		
Less harmful	197	0.67
Equal or more harmful	78	0.27
Don’t know	18	0.06
**Relative enjoyability of ENDS vs cigarettes**		
Less enjoyable	131	0.45
Equal or more enjoyable	161	0.55
**Cigarette craving reduction with ENDS**		
Not at all or a little	147	0.51
A lot or completely	140	0.49
	***Mean (SD)***	
**Irritability reduction** (ENDS)^a^	4.02 (1.76)	
**Taste liking** (ENDS)^a^	4.91 (1.66)	
**Satisfaction** (ENDS)^a^	4.43 (1.62)	
**Age** (years)	39.33 (9.82)	
	***n***	***Proportion***
**Gender**		
Male	97	0.32
Female	206	0.68
**Education level**		
Lower than or equal to high school	112	0.33
Higher than high school	191	0.63
**Racial/ethnic minoritized group status**		
Racial/ethnic minoritized group	82	0.27
Non-Hispanic White	221	0.73
	***Mean (SD)***	
**Cigarette withdrawal** (cravings)	8.82 (3.68)	
**Psychological distress**	8.79 (5.44)	
**Irritability reduction** (cigarettes)^a^	5.21 (1.63)	
**Taste liking** (cigarettes)^a^	4.04 (1.89)	
**Satisfaction** (cigarettes)^a^	5.29 (1.49)	

ENDS: electronic nicotine delivery systems.

a Responses ranged from 1= ‘not at all’ to 7= ‘extremely’, and were analyzed as a continuous predictor.

### Perceived relative harm of ENDS

At baseline, 27% of participants perceived ENDS equally or more harmful than cigarettes and 6% were uncertain of their relative harm. Compared to participants perceiving ENDS as equally or more harmful than cigarettes, those perceiving ENDS as less harmful had 83% lower adjusted odds (AOR=0.17; 95% CI: 0.05–0.60, p=0.006) of rejecting ENDS and returning to exclusive smoking as opposed to quitting smoking (switching exclusively to ENDS or quitting both ENDS and cigarettes) at follow-up at 1 month (Table 2, Model 1). Participants who were uncertain about the relative harm of ENDS had 97% lower adjusted odds of rejecting ENDS (AOR=0.03; 95% CI: 0.00–0.45, p=0.011) and 89% lower adjusted odds of being a concurrent user who primarily smokes cigarettes (AOR=0.11; 95% CI: 0.01–0.76, p=0.027) compared to quitting smoking. Perceived relative harm did not significantly distinguish other concurrent use patterns versus quitting smoking.

**Table 2:** Multinomial logistic regression of tobacco and ENDS use status at follow-up at 1 month on initial perception and user experience variables among recent initiators of ENDS at baseline, 2020–2021 (N=302)

	*Tobacco and ENDS use status (reference: Switchers/Quitters) ^a^*			
	*Rejectors*	*Primary smokers*	*Dual users*	*Primary vapers*
	*AOR (95% CI), p*	*AOR (95% CI), p*	*AOR (95% CI), p*	*AOR (95% CI), p*
**Model 1. ‘Is using electronic nicotine products less harmful, about the same, or more harmful than smoking regular cigarettes?’** (N=294)				
‘Much less harmful’ or ‘less harmful’ (vs ‘about the same’ or ‘more harmful’ or ‘much more harmful’)	**0.17 (0.05–0.60), 0.006**	0.29 (0.08–1.05), 0.058	0.45 (0.11–1.78), 0.255	1.78 (0.38–8.31), 0.463
‘Dontꞌ know’ (vs ‘about the same’ or ‘more harmful’ or ‘much more harmful’)	**0.03 (0.00–0.45), 0.011**	**0.11 (0.01–0.76), 0.027**	0.33 (0.04–2.72), 0.302	0.98 (0.11–0.08), 0.988
**Model 2. ‘How would you compare the experience of using electronic nicotine products to smoking regular cigarettes?’** (N=293)				
‘Less enjoyable’ (vs ‘equally enjoyable’ or ‘more enjoyable’)	**4.11 (1.45–11.65), 0.008**	2.53 (0.**96**–6.67), 0.060	1.31 (0.43–3.98), 0.629	0.90 (0.30–2.70), 0.855
**Model 3. ‘When I use electronic nicotine products, my cravings to smoke a cigarette are reduced…’** (N=293)^b^				
‘A little’ or ‘not at all’ vs ‘completely’ or ‘a lot’	**8.01 (2.72–23.62), <0.001**	**6.13 (2.32–16.17), <0.001**	1.64 (0.54–5.01), 0.387	1.53 (0.55–4.23), 0.414
**Model 4. ‘Did vaping make you feel less irritable?’** (N=293)^c^				
Less irritable	**0.49 (0.36–0.66), <0.001**	**0.62 (0.47–0.82), 0.001**	0.76 (0.56–1.03), 0.079	1.20 (0.87–1.66), 0.258
**Model 5. ‘Did your electronic nicotine product taste good?’** (N=293)^c^				
Taste good	**0.65 (0.47–0.91), 0.011**	0.90 (0.67–1.20), 0.472	1.17 (0.85–1.60), 0.335	1.16 (0.85–1.59), 0.358
**Model 6. ‘Was vaping satisfying?’** (N=293)^c^				
Satisfying	**0.45 (0.32–0.63), <0.001**	**0.60 (0.45–0.80), 0.001**	0.91 (0.67–1.23), 0.530	1.09 (0.79–1.51), 0.601

AOR: adjusted odds ratio.

a All models adjust for age, gender, race/ethnicity, education level, nicotine withdrawal, satisfaction with smoking cigarettes, experiencing less irritability when smoking, perceiving that cigarettes taste good, and mental health. AORs for covariates for each model are provided in Supplementary file Table S2.

b Not applicable/‘I do not have cravings to smoke a cigarette’ responses were handled as missing data.

c Responses ranged from 1= ‘not at all’ to 7= ‘extremely’, and were analyzed as a continuous predictor. Bolded coefficients are statistically significant at p<0.05.

### Enjoyability of ENDS relative to cigarettes

At baseline, 45% of participants experienced ENDS as less enjoyable than cigarettes, whereas the rest experienced them as equally or more enjoyable. Participants who experienced them as less enjoyable, compared to equally or more enjoyable, had more than four times the adjusted odds (AOR=4.11; 95% CI: 1.45–11.65, p=0.008) of discontinuing ENDS by the follow-up at 1 month, relative to switching exclusively to them or quitting both ENDS and smoking (Table 2, Model 2). Relative enjoyability was not significantly associated with odds of any concurrent use status relative to quitting smoking.

### Cigarette craving reduction with ENDS use

Among participants who reported craving cigarettes, 49% reported that using ENDS reduced their cigarette cravings a lot or completely. Compared to participants experiencing a lot or complete cigarette craving reduction when using ENDS, those who experienced no or a little craving reduction had more than eight times the adjusted odds (AOR=8.01; 95% CI: 2.72–23.62, p<0.001) of continuing exclusive smoking and rejecting ENDS. Similarly, those who experienced no or a little craving reduction had more than six times the adjusted odds (AOR=6.13; 95% CI: 2.32–16.17, p<0.001) of primarily smoking but concurrently using ENDS than quitting smoking at follow-up at 1 month (Model 3). The experience of craving reduction was not significantly associated with being a dual user or primary vaper relative to being a switcher/quitter.

### Irritability reduction with ENDS use

On average, participants reported a moderate reduction in irritability when using ENDS (mean irritability reduction=4.02 on a scale of 1 = ‘not at all’ to 7 = ‘extremely’) compared to using cigarettes (mean irritability reduction = 5.21). Each unit increase in irritability reduction was associated with a 51% reduction in the adjusted odds (AOR=0.49; 95% CI: 0.36–0.66, p<0.001) of rejecting ENDS and returning to exclusive smoking and a 38% lower adjusted odds (AOR=0.62; 95% CI: 0.47–0.82, p=0.001) of primarily smoking while continuing ENDS compared to quitting cigarettes (Model 4). The experience of irritability reduction when using ENDS was not significantly associated with being a dual user or primary vaper relative to being a switcher/quitter.

### Tastes good

Participants reported liking the taste of their ENDS (mean taste liking = 4.91 on a scale of 1 = ‘not at all’ to 7 = ‘extremely’, compared to 4.04 with cigarettes). Each unit increase in liking the taste of their ENDS was associated with a 35% reduction in the adjusted odds (AOR=0.65; 95% CI: 0.47–0.91, p=0.011) of rejecting ENDS by the follow-up at 1 month, relative to quitting smoking (Model 5). Liking the taste of their ENDS was not associated with being a concurrent user of cigarettes and ENDS relative to being a switcher/quitter.

### Satisfying

Overall satisfaction with ENDS was moderately high (mean satisfaction=4.43 on a scale of 1 = ‘not at all’ to 7 = ‘extremely’) but satisfaction was higher for cigarettes (5.29). Each unit increase in overall satisfaction with their ENDS was associated with a 55% reduction in the adjusted odds (AOR=0.45; 95% CI: 0.32–0.63, p<0.001) of rejecting ENDS and a 40% reduction in the adjusted odds (AOR=0.60; 95% CI: 0.45–0.80, p=0.001) of being a primary smoker at follow-up at 1 month, relative to quitting smoking (Model 6). Satisfaction with ENDS was not significantly associated with being a dual user or primary vaper relative to being a switcher/quitter.

## DISCUSSION

This study is among the first studies to examine smokers during the early critical period of ENDS initiation. Its results support our hypotheses that perceptions of and experiences with ENDS are predictive of short-term ENDS and smoking use patterns. Specifically, switchers/quitters and primary vapers were more satisfied with vaping and experienced greater craving reduction compared to rejectors and primary smokers. Results from our study show that established cigarette users separated into different smoking and ENDS use patterns based on perceived relative harm, perceived relative enjoyment from ENDS, and experiences such as cigarette craving reduction from ENDS use, irritability reduction with ENDS use, ENDS satisfaction, and ENDS taste within a relatively short period of time after initiating ENDS (i.e. 30–60 days). Overall results highlight that both switchers/quitters and primary vapers had overall more positive perceptions about and experiences with ENDS, including perceiving ENDS as less harmful and equally or more enjoyable than cigarettes. In contrast, primary smokers and rejectors had more negative views, with about 60% of primary smokers and 90% of rejectors perceiving ENDS as less enjoyable than cigarettes.

These findings offer explanations on why people who smoke differ in switching or not switching to exclusive vaping during a short period of time following initiation of ENDS use. Most studies tracking smoking and ENDS use have assessed patterns at follow-up at 1 year^1^. Results from our study regarding perceived harm and satisfaction are consistent with this literature but add unique information about the early period of initiating ENDS. For example, other studies have found that individuals who believe ENDS are safer than combustible tobacco products are more likely to try ENDS, and those using ENDS compared to cigarettes are more likely to report a desire to quit cigarettes^22,33^. Similarly, those who are more satisfied with ENDS are more likely to continue using ENDS and decrease cigarette consumption^4,17^. In our study, participants who positively rated the perceptions and experiences of ENDS were more often the switchers/quitters rather than the rejectors or primary smokers at follow-up at 1 month. While previous studies have examined similar perceptions and measures of satisfaction among current and former ENDS users, those studies focused largely on established ENDS users or youth populations^1,17^. The current study uniquely examined these factors as well as additional factors among established cigarette smokers during the critical period of initiating (or re-initiating) ENDS use. Additionally, most prior follow-ups of smokers initiating use of ENDS contrast rejectors and switchers/quitters, but combine primary smokers and primary vapers into the dual use category. While our study sought to recruit only established smokers who would qualify as dual users, as typically defined in many past studies, our dual users (similar levels of use of cigarettes and ENDS) represented only 13% of our participants at the follow-up at 1 month. As noted above, our results show that primary smokers are significantly different in their perceptions of and initial experiences with ENDS. Thus, these results suggest that much of the published literature about dual users may crudely characterize patterns of use of ENDS among smokers.

Our sample of established smokers was not recruited based upon intentions to use ENDS to quit smoking. However, evidence shows that smokers often initiate using ENDS as an alternative to smoking or to quit smoking completely^4-6^. In the analyses of a US-representative sample of smokers who initiated ENDS use to help quit smoking, concerns have been raised about rates of relapse back to exclusive smoking^9^. Among our smokers, the rating of the degree to which using ENDS was perceived to reduce cigarette craving and irritability was highly predictive of those who had not completely switched to vaping at the follow-up at 1 month, namely the rejectors and primary smokers. Individuals who try to quit smoking may experience nicotine withdrawal symptoms, including but not limited to cigarette cravings, irritability, difficulty concentrating, and anxiety^34^. Research has found that different environmental reminders can trigger cigarette cravings, and cigarette cravings have been found to be associated with smoking relapse among individuals trying to quit cigarettes^34,35^. When individuals go without smoking, it can lead to cigarette cravings and irritability, which temporarily seem to be relieved by smoking. Therefore, if ENDS use is unable to reduce the cravings and irritability of individuals trying to quit smoking cigarettes, evidence suggests they are more likely to either reject ENDS or use some ENDS but primarily smoke cigarettes.

Results highlight that perceived harm, enjoyment, cigarette craving reduction, irritability reduction, ENDS satisfaction, and ENDS taste are associated with different use patterns at the follow-up at 1 month. Our findings show how these factors could influence ENDS users’ trajectories (either to primary or exclusive use of cigarettes or switching completely to ENDS and/or quitting all tobacco products), thus impacting public health outcomes, including likelihood of ongoing tobacco use and increased risk of tobacco-related illnesses. Prior research indicates that whether ENDS could act as a disruptive technology was uncertain as users were less pleased with these products compared to cigarettes^27^. In our study, ENDS may act as a disruptive technology for people with more positive views of these factors, such as switchers/quitters and primary vapers, but not for those with more negative perceptions and experiences. A policy agenda for ENDS in the US has been proposed^26^. The proposed policy agenda emphasizes the importance of product design and related communication about the relative harm of ENDS compared to cigarettes^26^. Recent research also highlighted the importance of ENDS product changes on how perceptions of harm develop among those who continue to use ENDS and those that discontinue use over time^36^. With advancements and changes in ENDS products, it is necessary to continue examining the impact of these changing ENDS products on cigarette consumption and patterns of use of ENDS. The results from this study offer important insights into various factors which appear important in monitoring, if the changes in ENDS products could improve their potential as a disruptive technology. However, it is still early in these users’ trajectories among our participants, and further data are needed to confirm how the baseline and changing perceptions and experiences with ENDS over time will predict longer term patterns of rejecting ENDS or a step toward exclusive use of ENDS or quitting both ENDS and smoking. Additional follow-up data should provide useful evidence that can assist in the development of policies related to product regulations and communications promoting a positive role of ENDS in reducing tobacco-related disease^10,26^. Additionally, these results suggest that longitudinal surveys to evaluate policy relevant changes in smoking behavior assess the categories of ENDS use, as suggested by Borland et al.^26,28^.

### Limitations

There are some limitations in this study. First, our sample is not nationally representative of the United States. The findings may not reflect national patterns of use, and subsample sizes for racial/ethnic minoritized groups were relatively small. The small sample size limited our ability to conduct sex stratified analyses, necessitated collapsing of levels for some covariate, predictor, and outcome variables in our analyses, and adjust for other factors; thus, residual confounding may exist. The sample’s sociodemographic characteristics are relatively homogenous, which may limit generalizability. In addition, we only had data on the frequency of use of cigarettes and ENDS in the past week to determine use patterns, and we acknowledge that other measures, such as the amount of consumption of cigarettes or ENDS, may have produced different use pattern groups.

## CONCLUSIONS

Perceived harm, enjoyment, cigarette craving reduction, irritability reduction, vaping satisfaction, and vape taste are necessary factors to consider among recent and new ENDS initiators’ use patterns and trajectories to cigarette cessation. Results demonstrate that ENDS initiators are separating into different use patterns based on several factors within a relatively short period of time. Future research should further examine perception factors as they might limit realworld use of ENDS to completely substitute for cigarette smoking.

## Supplementary Material



## Data Availability

The data supporting this research are available from the following sources: https://doi.org/10.57709/ed4q-d040
