# Supplementary material for: Examining longitudinal associations between initial perceptions and experiences with electronic nicotine delivery system (ENDS) use and use patterns among adults who smoke and recently initiated ENDS
Source: Tob Induc Dis. 2024 Sep 27;22:10.18332/tid/193009. doi: 10.18332/tid/193009 (PMC11427937; doi:10.18332/tid/193009)
Supplement: Supplementary file 1 [file TID-22-164-s1.pdf]

Table S1. Characterization of cigarette and ENDS use patterns at baseline survey, 2020-2021 (N=303)

Vaping Status	Smoking Status		
	Not at all	Some days	Every day
Not at all	No current use (n=2)	Rejectors (n=0)	Rejectors (n=19)
Some days	Switchers (n=4)	Dual Users (n=0)	Primary Smokers (n=134)
Every day	Switchers (n=28)	Primary Vapers (n=58)	Dual Users (n=58)

Note. All participants at screener interview (n=303) were recent or current established cigarette smokers who had recently initiated or re-initiated use of ENDS. Dual Users had an equal frequency of past week vaping and smoking cigarettes. Primary-Smokers had a greater frequency of smoking cigarettes in the past week than frequency of past week vaping. Primary-Vapers had a greater frequency of vaping in the past week than smoking cigarettes.

Table S2. Full results for multinomial logistic regression of tobacco and ENDS use status at 1-month follow-up on initial perception and user experience variables among recent initiators of ENDS at baseline, 2020-2021 (N=294)

	**Tobacco and ENDS Use Status (reference = Switchers & Quitters)[a]**			
	**Rejectors**	**Primary Smokers**	**Dual Users**	**Primary Vapers**
	**aOR (95% CI), p-value**	**aOR (95% CI), p-value**	**aOR (95% CI), p-value**	**aOR (95% CI), p-value**
***Model 1: Is using electronic nicotine products less harmful, about the same, or more harmful than smoking regular cigarettes?* (n=294) [b]**				
'Much less harmful' or 'less harmful' (vs. 'about the same' or 'more harmful' or 'much more harmful')	**0.17 (0.05-0.60), .006**	0.29 (0.08-1.05), .058	0.45 (0.11-1.78), .255	1.78 (0.38, 8.31), .463
"Don't know" (vs. 'about the same' or 'more harmful' or 'much more harmful')	**0.03 (0.00-0.45), .011**	**0.11 (0.01-0.76), .027**	0.33 (0.04-2.72), .302	0.98 (0.11-0.08), .988
Age	1.00 (0.95-1.04), .802	**1.04 (1.00-1.09), .045**	1.02 (0.97-1.08), .354	0.99 (0.95-1.04), .730
Male	0.40 (0.15-1.07), .068	**0.25 (0.10-0.62), .003**	0.49 (0.17-1.41), .185	0.66 (0.27-1.64), .374
White, non-Hispanic	1.68 (0.55-5.11), .361	1.96 (0.67-5.68), .217	0.95 (0.29-3.05), .924	1.50 (0.52-4.35), .454
Greater than high school education	0.98 (0.38-2.54), .971	1.18 (0.50-2.79), .707	1.06 (0.37-3.02), .918	0.74 (0.29-1.84), .516
Satisfaction (cigarettes)	1.06 (0.68-1.65), .803	1.04 (0.71-1.54), .839	1.04 (0.67-1.60), .873	0.87 (0.56-1.35), .525

Taste liking (cigarettes)	1.03 (0.77-1.39), .839	1.20 (0.91-1.58), .195	1.22 (0.89-1.67), .205	0.97 (0.71-1.34), .856
Irritability reduction (cigarettes)	0.86 (0.61-1.17), .304	0.80 (0.60-1.06), .123	0.86 (0.60-1.23), .405	0.94 (0.70-1.30), .707
Cigarette withdrawal (cravings)	**0.75 (0.65-0.86), <.001**	**0.78 (0.69-0.88), <.001**	**0.80 (0.70-0.92), .001**	0.93 (0.82-1.05), .238
Psychological distress	0.98 (0.91-1.09), .933	1.01 (0.93-1.10), .764	1.03 (0.94-1.13), .542	0.93 (0.85-1.02), .140
Model 2: How would you compare the experience of using electronic nicotine products to smoking regular cigarettes? **(n=293)**				
'Less enjoyable' (vs. 'equally enjoyable' or 'more enjoyable')	**4.11 (1.45-11.65), .008**	2.53 (0.96-6.67), .060	1.31 (0.43-3.98), .629	0.90 (0.30-2.70), .855
Age	0.99 (0.94-1.03), .487	1.04 (.99-1.08), .097	1.02 (0.97-1.08), .385	0.99 (0.95-1.05), .908
Male	0.54 (0.20-1.43), .213	0.31 (0.12-0.81), .017	0.55 (0.19-1.55), .258	0.71 (0.28-1.77), .460
White non-Hispanics	1.42 (0.49-4.15), .516	1.75 (0.61-5.02), .301	0.92 (0.30-2.684, .886	1.55 (0.56-4.33), .399
Greater than high school education	0.83 (0.33-2.08), .692	1.11 (0.48-2.56), .815	1.04 (0.38-2.87), .942	0.79 (0.33-1.89), .598
Satisfaction (cigarette)	0.86 (0.55-1.35), .504	0.90 (0.61-1.33), .600	0.95 (0.61-1.48), .811	0.88 (0.58-1.35), .563
Taste liking (cigarette)	1.07 (0.79-1.46), .653	1.24 (0.94-1.63), .130	1.24 (0.91-1.69), .168	0.98 (0.72-1.33), .877
Irritability reduction (cigarette)	0.91 (0.66-1.24), .531	0.85 (0.64-1.11), .230	0.90 (0.65-1.25), .532	0.93 (0.70-1.24), .624

Cigarette withdrawal (cravings)	**0.77 (0.67-0.88), <.001**	**0.80 (0.71-0.90), <.001**	**0.82 (0.72-0.93), .002**	0.93 (0.84-1.05), 222
Psychological distress	0.96 (0.87-1.05), .392	0.99 (0.91-1.07), .840	1.02 (0.94-1.12), .629	0.94 (0.85-1.03), .152
Model 3: When I use electronic nicotine products, my cravings to smoke a cigarette are reduced… **(n=293)**[b]				
'Completely' or 'a lot' vs. 'a little' or 'not at all'	**8.01 (2.72-23.62), <.001**	**6.13 (2.32-16.17), .001**	1.64 (0.54-5.01), .079	1.53 (0.55-4.23), .258
Age	1.00 (0.95-1.05), .946	1.05 (1.01-1.09), .130	1.03 (0.98-1.08), .467	0.99 (0.95-1.04), .909
Male	0.57 (0.22-1.52), .263	**0.35 (0.14-0.91), .027**	0.55 (0.19-1.57), .363	0.71 (0.29-1.76), .361
White non-Hispanics	1.47 (0.52-4.17), .473	1.79 (0.62-5.16), .331	0.92 (0.30-2.82), .850	1.52 (0.55-4.21), .443
Greater than high school education	0.89 (0.34-2.34), .811	1.14 (0.47-2.75), .487	1.03 (0.37-2.88), .836	0.77 (0.31-1.89), .561
Satisfaction (cigarette)	0.93 (0.58-1.49), .756	0.94 (0.63-1.41), .807	0.97 (0.64-1.48), .850	0.90 (0.60-1.36), .490
Taste liking (cigarette)	0.99 (0.71-1.39), .979	1.17 (0.86-1.59), .119	1.22 (0.89-1.67), .185	0.96 (0.70-1.32), .951
Irritability reduction (cigarette)	0.94 (0.69-1.29), .712	0.87 (0.66-1.15), .937	0.89 (0.65-1.24), .948	0.92 (0.70-1.22), .372
Cigarette withdrawal (cravings)	**0.80 (0.69-0.92), .002**	**0.83 (0.74-0.94), <.001**	**0.82 (0.72-0.94), .001**	0.94 (0.84-1.06), .380
Psychological distress	0.97 (0.88-1.07), .541	1.00 (0.92-1.09), .895	1.03 (0.94-1.12), .651	0.93 (0.86-1.02), .171
Model 4: Did vaping make you feel less irritable? **(n=293)**[c]				

Less irritable (ENDS)	**0.49 (0.36-0.66), <.001**	**0.62 (0.47-0.82), <.001**	0.76 (0.56-1.03), .065	1.20 (0.87-1.66), .214
Age	0.98 (0.94-1.03), .485	1.03 (0.99-1.08), .045	1.02 (0.97-1.07), .251	.99 (0.95-1.05), .737
Male	0.63 (0.22-1.76), .378	0.34 (0.13-0.88), .057	0.62 (0.22-1.75), .552	0.65 (0.25-1.65), .497
White non-Hispanics	1.31 (0.42-4.09), .640	1.71 (0.58-5.00), .347	0.89 (0.28-2.89), .788	1.51 (0.53-4.29), .637
Greater than high school education	1.11 (0.42-2.92), .835	1.36 (0.57-3.24), .513	1.12 (0.39-3.17), .857	0.77 (0.31-1.88), .527
Satisfaction (cigarette)	0.95 (0.62-1.45), .799	0.95 (0.65-1.40), .705	0.96 (0.62-1.49), .747	0.85 (0.54-1.35), .477
Taste liking (cigarette)	1.08 (0.79-1.46), .640	1.25 (0.95-1.65), .148	1.23 (0.90-1.68), .225	0.99 (0.72-1.36), .802
Irritability reduction (cigarette)	1.16 (0.85-1.58), .344	1.01 (0.76-1.35), .775	1.01 (0.73-1.40), .810	0.87 (0.65-1.17), .491
Cigarette withdrawal (cravings)	**0.73 (0.63-0.85), <.001**	**0.77 (0.67-0.87), <.001**	**0.80 (0.69-0.91), .001**	0.95 (0.83-1.07), .378
Psychological distress	0.97 (0.88-1.06), .497	0.99 (0.91-1.08), .688	1.02 (0.93-1.12), .804	0.93 (0.85-1.03), .124
Model 5: Did your electronic nicotine product taste good? **(n=293)**[c]				
Taste good (ENDS)	**0.65 (0.47-0.91), .011**	0.90 (0.67-1.20), .472	1.17 (0.85-1.60), .335	1.16 (0.85-1.60), .358
Age	0.99 (0.94-0.91), .552	1.04 (0.99-1.08), .074	1.03 (0.98-1.09), .266	1.00 (0.95-1.05), .996
Male	0.48 (0.18-1.28), .142	**0.28 (0.11-0.71), .007**	0.50 (0.18-1.38), .183	0.66 (0.26-1.65), .375
White non-Hispanic	1.34 (0.46-3.89), .588	1.78 (0.63-5.01), .278	0.93 (0.29-2.89), .900	1.53 (0.55-4.26), .416
Greater than high school education	1.12 (0.44-2.84), .810	1.22 (0.52-2.87), .643	0.99 (0.35-2.81), .980	0.74 (0.30-1.83), .518

Satisfaction (cigarettes)	0.95 (0.58-1.53), .825	0.95 (0.63-1.41), .780	0.92 (0.35-2.81), .704	0.85 (0.55-1.31), .459
Taste liking (cigarettes)	1.11 (0.80-1.54), .533	1.26 (0.95-1.68), .114	1.26 (0.91-1.65), .159	0.98 (0.71-1.35), .876
Irritability reduction (cigarettes)	0.92 (0.67-1.27), .629	0.86 (0.65-1.12), .254	0.89 (0.64-1.22), .460	0.92 (0.70-1.21), .571
Cigarette withdrawal (cravings)	**0.74 (0.64-0.85), <.001**	**0.78 (0.69-0.89), <.001**	**0.82 (0.72-0.94), .003**	0.94 (0.83-1.06), .305
Psychological distress	0.98 (0.89-1.07), .597	1.00 (0.92-1.07), .972	1.03 (0.94-1.12), .554	0.94 (0.86-1.03), .158
***Model 6: Was vaping satisfying?* (n=293)[c]**				
Satisfying	**0.45 (0.32-0.63), <.001**	**0.60 (0.45-0.80), .001**	0.91 (0.67-1.23), .530	1.09 (0.79-1.51), .601
Age	0.99 (0.94-1.04), .585	1.04 (0.99-1.08), .089	1.02 (0.98-1.08), .341	0.99 (0.95-1.05), .893
Male	0.57 (0.21-1.56), .273	0.33 (0.13-0.85), .052	0.54 (0.20-1.50), .238	0.69 (0.28-1.72), .435
White non-Hispanic	1.22 (0.39-3.78), .725	1.60 (0.55-4.70), .392	0.89 (0.28-2.79), .836	1.56 (0.56-4.46), .408
Greater than high school education	0.97 (0.36-2.59), .955	1.16 (0.48-2.79), .739	1.06 (0.37-3.02), .914	0.79 (0.32-1.92), .596
Satisfaction (cigarettes)	0.98 (0.62-1.57), .946	1.02 (0.68-1.51), .933	0.97 (0.63-1.48), .878	0.86 (0.56-1.32), .496
Taste liking (cigarettes)	1.13 (0.82-1.57), .457	1.27 (0.95-1.68), .105	1.24 (0.91-1.69), .172	0.98 (0.72-1.34), .911

Irritability reduction (cigarettes)	1.02 (0.74-1.42), .886	0.92 (0.69-0.89), .545	0.93 (0.68-1.27), .626	0.93 (0.71-1.22), .584
Cigarette withdrawal (cravings)	**0.75 (0.65-0.87), <.001**	**0.79 (0.69-0.90), <.001**	**0.82 (0.73-0.93), .002**	0.93 (0.83-1.05), .244
Psychological distress	0.98 (0.89-1.08), .669	1.01 (0.93-1.09), .906	1.03 (0.94-1.12), .578	0.94 (0.86-1.02), .135

ENDS: electronic nicotine delivery systems. Bolded coefficients are statistically significant at $p < .05$.

[a] Switchers and Quitters were combined to form a single category 'Switchers and Quitters" due to small cell size.

[b] "Not applicable – I do not have cravings to smoke a cigarette" responses were handled as missing data.

[c] Responses ranging from 1-7 ('Not at all' to 'Extremely') and analyzed as a continuous predictor.

© 2024 Mavreles Ogrodnick M. et al.
